# Thiamin (Vitamin B_1_) – A scoping review for Nordic Nutrition Recommendations 2023

**DOI:** 10.29219/fnr.v67.10290

**Published:** 2023-11-13

**Authors:** Hanna Sara Strandler, Tor A. Strand

**Affiliations:** 1 Swedish Food Agency, Uppsala, Sweden; 2 Department of Global Public Health and Primary Care, Sykehuset Innlandet HF, University of Bergen, Bergen, Norway

**Keywords:** thiamine, vitamin B_1_, vitamins, recommendations

## Abstract

Only a few studies have explored relationships between thiamine intake and function, and a few studies have examined the effects of supplements on various clinical or biochemical outcomes. None of these studies, however, makes a useful contribution to understanding requirements in healthy populations. The requirement of thiamine relates to energy and carbohydrate intake. Clinical signs of deficiency have been observed at intakes below 0.5 mg/day, which corresponds to 0.05 mg/MJ. In other studies, thiamine excretion in the urine and normalisation of enzyme activity were normalised at intakes of 0.07–0.08 mg/MJ. The lower limit of intake thus estimates at 0.05 mg/MJ. It has not been possible to set a safe upper intake level for thiamine due to a lack of data.

Studies on pregnant and lactating women indicate a higher requirement as assessed by biochemical parameters. A few studies indicate that thiamine utilisation is impaired among elderly subjects.

## Popular scientific summary

- Thiamin (vitamin B_1_) is a water-soluble vitamin, essential for the metabolism of glucose, amino acids and lipids.
- Thiamin requirements are related to energy and carbohydrate intake.
- Transketolase activity in erythrocytes and free thiamine in blood or erythrocytes are biomarkers of thiamine status.
- Deficiency manifests as beriberi or Wernicke-Korsakoff syndrome.
- Cereals and cereal products, meat and meat products, and milk and dairy products are major dietary sources of thiamine in the Nordic and Baltic populations.

Like the other B-vitamins, thiamine is necessary for the function of enzymes in human metabolism but cannot be synthesised in the human body. The predominant form of thiamine, thiamine diphosphate (ThDP), functions as a coenzyme in reactions involved in the metabolism of glucose, amino acids and lipids (1–3).

Unlike the other B-vitamins, the requirement of thiamine depends on the non-lipid energy intake (4). The extent of substrate utilisation does not usually affect coenzymes. A possible explanation why thiamine is affected may be that ThDP, while bound to a ThDP-dependent enzyme, becomes unstable and thereby susceptible for deterioration (5). The need of thiamine increases with increased metabolic rate, for example, during physical activity, pregnancy and lactation. Thiamin requirements also increase in conditions such as hyperthyroidism and protracted fever or due to genetic disorders (3, 6, 7).

Transketolase is one of the enzymes in the metabolic processes that need ThDP as a coenzyme (8, 9). The measurement of the enzymatic activity of trans-ketolase in the erythrocytes (ETKAC) is therefore useful as an indicator of thiamine status, while the most responsive biomarker of thiamine intake is plasma thiamine (10).

Severe thiamine deficiency causes beriberi, a disease with a complex representation of both neurological and cardiovascular disorders. Beriberi became frequent in the 19th century in countries with rice as a staple food and main part of the diet with the introduction of a practice of polishing the rice. Polishing removes thiamine from rice since it is located in the husk. In search for a remedy for beriberi, recognition of the benefits of a varied diet and the discovery of thiamine eventually enabled dietary prevention and treatment. However, beriberi is still a concern globally for various reasons (11). Even in countries with supposedly sufficient diets, studies indicate that subclinical deficiency may be more prevalent than could be expected (7). Wernicke-Korsakoff syndrome, the third most common cause of dementia after Alzheimer’s disease, is a condition of severe brain function impairment caused by thiamine deficiency related to chronic alcohol abuse (7).

Synthesis of thiamine occurs in plants, protozoans and fungi, whereas animals and humans need vitamin from the diet. Foods with relatively high content are most meats, eggs, fruits and vegetables. Foods rich in thiamine content are yeast, organ foods and lean pork as well as pulses, nuts, wheat bran, oatmeal and whole-grain cereals, while milk has a relative low content of thiamine. Regardless of content, the amount of food consumed determines the intake of thiamine. Since thiamine is the most heat-labile vitamin, processing the food causes some thermal destruction of thiamine (6).

This scoping review aims to describe the totality of evidence for the role of thiamine for health-related outcomes as a basis for setting and updating dietary reference values (DRVs) in the Nordic Nutrition Recommendations 2023 (Box 1).

Box 1. Background papers for Nordic Nutrition Recommendations 2023.

- This paper is one of many scoping reviews commissioned as part of the Nordic Nutrition Recommendations 2023 (NNR2023) project (12).
- The papers are included in the extended NNR2023 report, but, for transparency, these scoping reviews are also published in Food & Nutrition Research.
- The scoping reviews have been peer reviewed by independent experts in the research field according to the standard procedures of the journal.
- The scoping reviews have also been subjected to public consultations (see report to be published by the NNR2023 project).
- The NNR2023 committee has served as the editorial board.
- While these papers are a main fundament, the NNR2023 committee has the sole responsibility for setting dietary reference values in the NNR2023 project.

## Methods

This review follows the protocol developed within the Nordic Nutrition Recommendations 2023 (NNR2023) project (12). The sources of evidence used in the scoping review follow the eligibility criteria described in the paper ‘The Nordic Nutrition Recommendations 2022 – Principles and methodologies’ (13). No de novo NNR2023 systematic reviews relevant for this scoping review were conducted (14). The main literature search for this review was performed on April 6, 2021 in MEDLINE with a search string: (thiamine[MeSH Terms] OR Thiamin[Title]) AND review[Publication Type] AND (“2011”[Date - Publication] : “3000”[Date - Publication]) AND humans[Filter]. The number of hits was 166. Based on the title, 12 articles were picked up, of which three was considered as relevant based on the full article. We also identified relevant literature for this review via ‘snowballing’/citation chasing that was relevant for the background information. No strong evidence was identified in scientific literature since 2012 that likely would cause a change in DRVs. Neither any topic related to a substantial health concern in the Nordic or Baltic countries was identified. An updated search was performed on August 1, 2022. The scoping review authors and the NNR committee are aware of these publications; however, the information in these publications does not change the judgement regarding the setting of DRVs for thiamine.

## Physiology

**Fig. 1:**
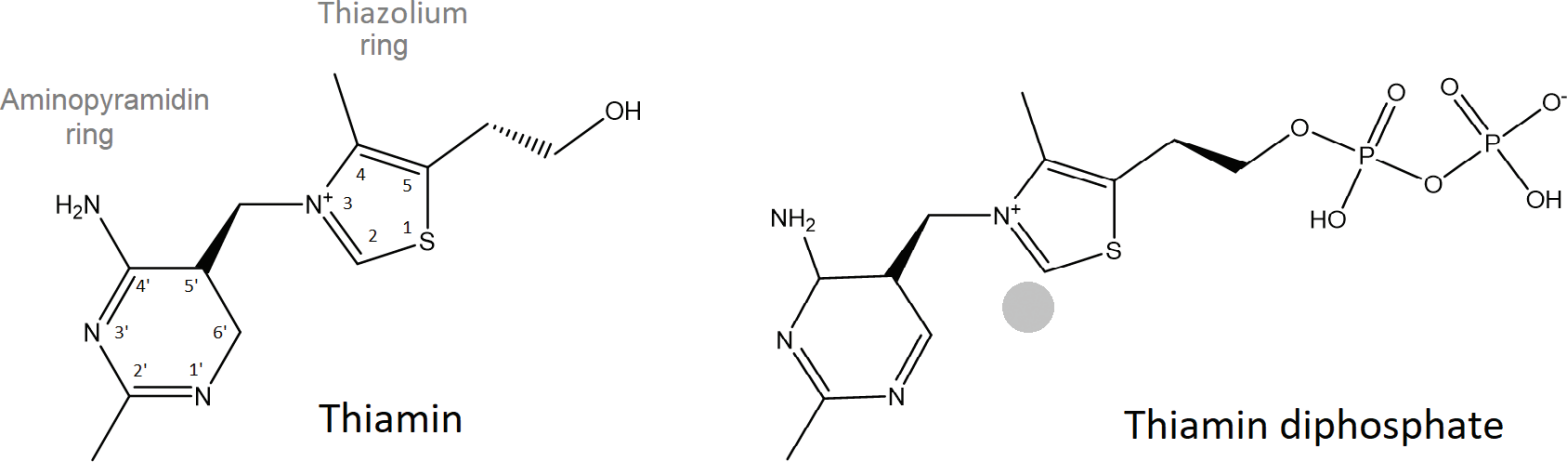
Thiamin and thiamin diphosphate (ThDP) structural formulas (16). The active centre of the ThDP is marked with (17, 18).

Thiamin is a water-soluble vitamin with molecular formula of C_12_H_17_N_4_OS+, molecular mass of 265.35 g/mol and a structure of substituted pyrimidine and thiazole rings joined by a methylene bridge (Fig. 1). The thiazole heterocycle ring is the active centre directly involved in catalysis by enzymes using ThDP as coenzyme. ThDP is tightly but not covalently bound to the apoenzyme. The aromatic pyrimidine heterocycle aids in catalysis and is needed for recognition by the enzyme active site (Fig. 1) (7).

Vitamin B_1_ is a generic term for substances with vitamin activity, the IUPAC name 3-[(4-amino-2-methylpyrimidin-5-yl) methyl]-4-methyl-1,3-thiazol-3-ium-5-yl]ethanol (15).

Besides the free form of thiamine, three phosphorylated forms are present in nature. The most common form in living tissues is ThDP (Fig. 1), also referred to as thiamine pyrophosphate (TPP). In animal tissues, small amounts of monophosphate (ThMP) and triphosphate (ThTP) can also be present. Other biologically active forms found in human tissue are adenosine thiamine triphosphate (ATTP) (19) and 2-(1-hydroxyethyl-thiamine) (HET). The latter, an intermediate substance in oxidative decarboxylation of pyruvate (18), may also be considered as a thiamine source from food (20). Substances approved for use in supplementation are salts of thiamine, thiamine chloride and thiamine mononitrate (21).

Thiamin is essential for the utilisation of carbohydrates of amino acid lysine and branched-chain amino acids as well as of 2-hydroxy fatty acids (8, 9). ThDP functions as a coenzyme for enzymes involved in oxidative decarboxylation or transketolation, facilitating cleavage or formation of a C-C bond directly next to a carbonyl group by proton substitution at carbon 2 in the thiazolium ring (Fig. 1) (2). ThTP is involved in nerve and possibly muscle function (2, 8).

**Fig. 2:**
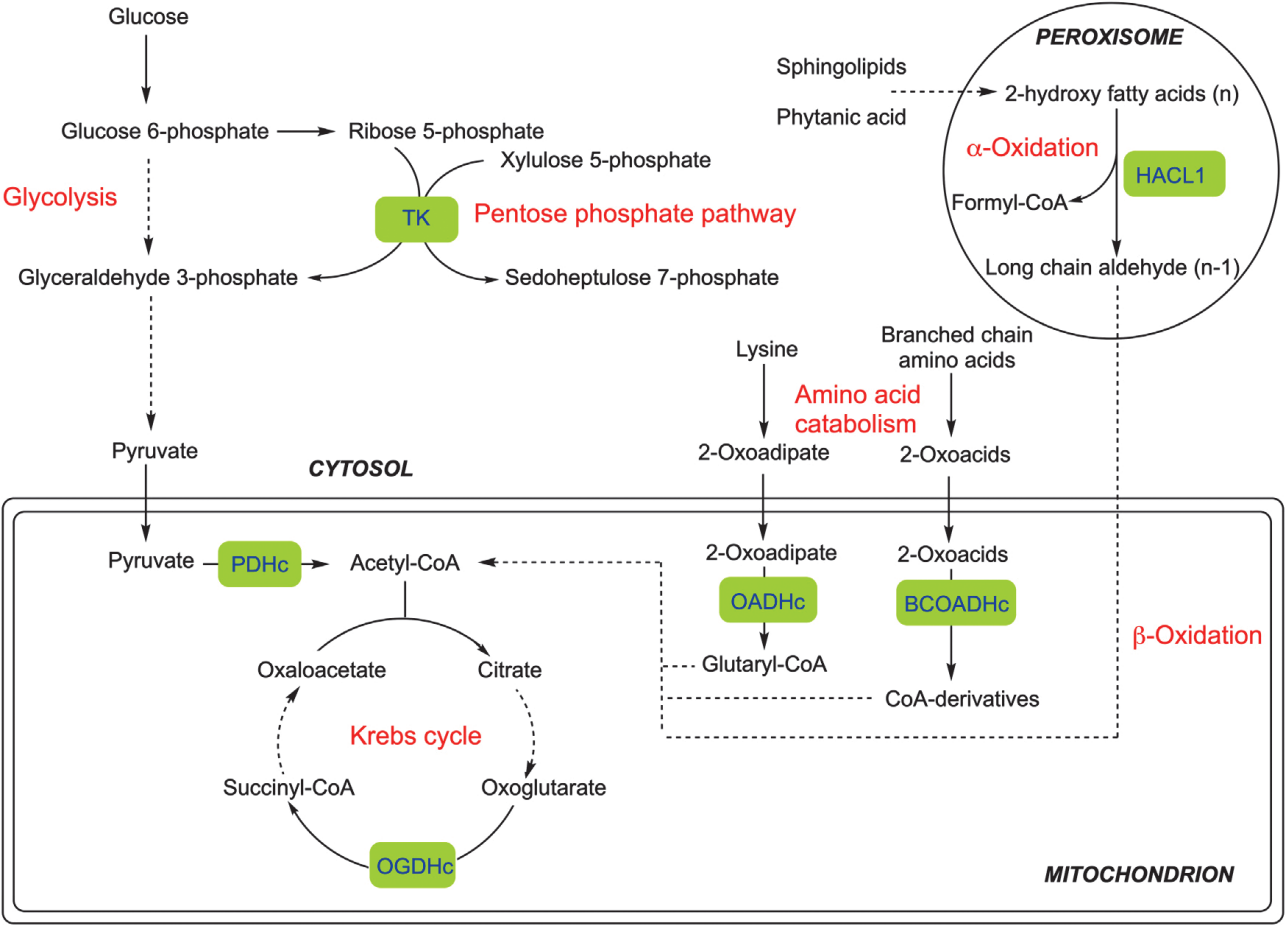
Role of ThDP-dependent enzymes in cellular metabolism. Thiamin-dependent enzyme is indicated in a green box. BCOADHc, branched-chain 2-oxoacid dehydrogenase complex; HACL1, 2-hydroxyacyl-CoA lyase 1; OADHc, 2-oxoadipate dehydrogenase complex; OGDHc, 2-oxoglutarate dehydrogenase complex; PDHc, pyruvate dehydrogenase complex; TK, transketolase. Used with permission, Bettendorf (2).

The role of ThDP as a cofactor is described in Fig. 2. In the cytosol, ThDP is needed by transketolase in the pentose and glycolytic pathways (2, 22). In the mitochondria, multi-enzyme complexes with thiamine-dependent enzymes are the pyruvate dehydrogenase multi-enzyme complex, which enable the conversion of pyruvate to acetyl-CoA, and α-ketoglurate dehydrogenate, also called oxoglutarate dehydrogenase, which catalyse the conversion of α-ketoglurate to succinyl CoA. Both of these reactions also require riboflavin (FAD) and niacin (NAD^+^) as well as Mg^2+^, lipoic acid and coenzyme-A. Multi-enzyme complexes are involved in amino acid catabolism. Leucine, isoleucine and valine undergo catabolism in the branched-chain 2-oxoacid dehydrogenase complex, and lysine the 2-oxoadipate dehydrogenase complex. In the peroxisome, 2-hydroxyacyl-CoA lyase enables the conversion of 2-hydroxy fatty acids to long chain aldehydes.

Thiamin in foods from plants is present in the free form, while in foods from animal origin, it is mainly in phosphorylated forms. Prior to absorption, phosphorylated forms are converted to free thiamine by phosphatases in the intestinal lumen (23, 24). Absorption of free thiamine takes place in the lumen of duodenum and proximal jejunum by a saturable carrier-mediated mechanism. Passive diffusion also takes place at high intakes, intakes that result in lumen concentrations above 2.5 µmol (25, 26). Thiamin can be obtained from the normal bacterial microflora of the large intestine and is absorbed in that region of the gut (24), but the quantitative importance of this source is uncertain. Studies with ^14^C-labelled thiamine in young men (27) showed that more than 95% of the vitamin was absorbed at intakes of 1–2 mg/day. At intakes above 5 mg/day, the relative absorption rapidly decreases (28). Besides genetic disorders, absorption may be impaired by alcohol or, to a much lesser extent, by anti-thiamine factors (1). Thiamin can be destroyed by cleavage of the methylene bridge by sulphites added for food preservation or by oxidation of the thiazole ring by thermo-stable substances in plants. These substances, polyphenols and hydroxylated substances of cinnamic acid, flavonoids and tannins are present in a variety of fruits, vegetables and berries as well as tea, coffee and rice bran (6). Thermo-labile enzymes, thiamineases, can destroy thiamine if the food is not cooked. Hence, regular consumption of raw or fermented freshwater fish, raw shellfish, ferns or insects can cause impaired thiamine intake (7).

**Fig. 3:**
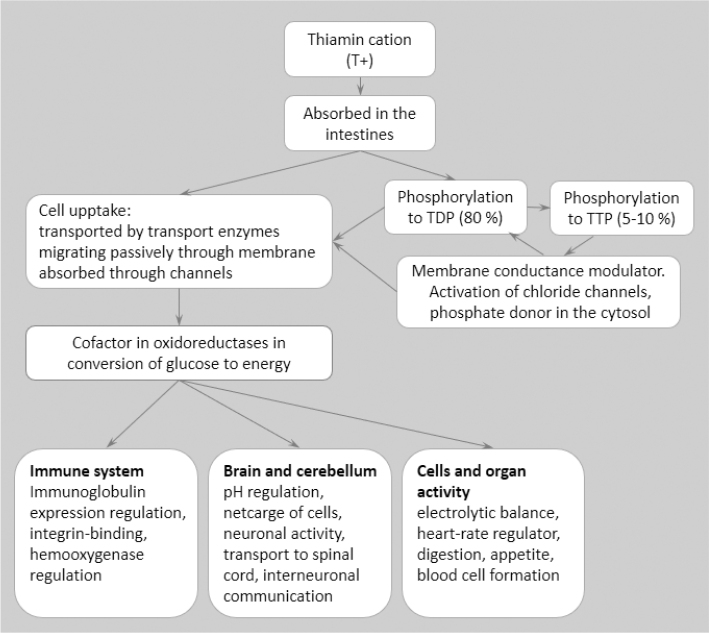
Physiological functions of thiamine in tissues and organs, modified from Manzetti (29).

**Fig. 4:**
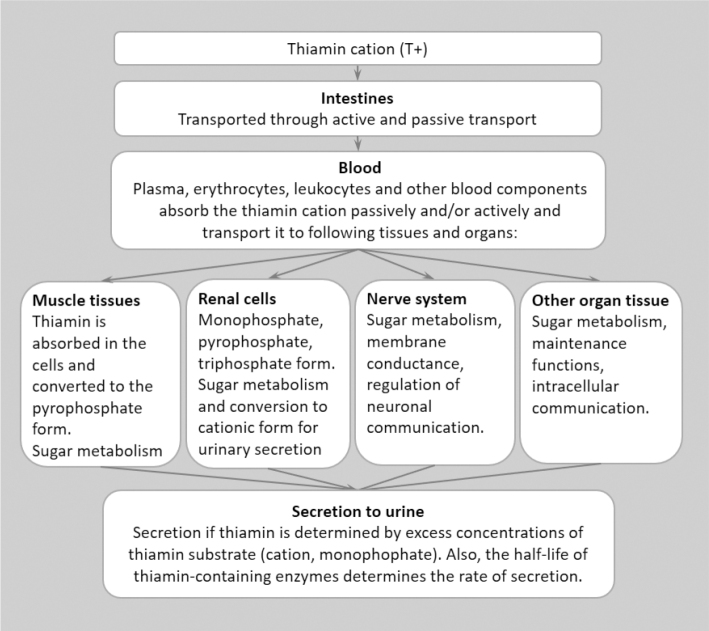
Physiological fate of thiamine in tissues and organs, modified from Manzetti (29).

After absorption, thiamine is transported to the liver and other organs in the body by the blood, and physiological functions and fate in different organs are described in Figs. 3 and 4. Plasma carries a lower amount as free thiamine, ThMP or protein-bound ThDP. The major part, about 80%, is captured by high-affinity transporters into erythrocytes and retained there by phosphorylation, mainly not only to ThDP but also further to ThTP, Fig. 3 (7, 19, 29).

Cells in other organs and tissues also use active transport for thiamine uptake. Transporters of thiamine belong to the solute carrier group of membrane transport proteins. ThTR1 and ThTR2, encoding genes SLC19A2 and SLC19A3, aid transport to systemic tissues, the latter intestinal thiamine absorption (3). Other transporters involved are reduced folate carrier protein, organic cation transporter and mitochondrial ThDP transporter (2). The vitamin is retained to function within the cell by phosphorylation and thiamin is released for transport in the body by dephosphorylation. Enzymes in the cell, TPP kinase or thiamin kinase, phosphorylate and thiaminepy-rophosphatease or thiamine triphosphatease, dephosphorylate. (1, 29).

The total body pool of thiamine in an adult is about 30 mg, and most of this is in the muscles and liver (9, 27). The metabolism of thiamine in the body is relatively fast, and the half-life of ^14^C-labelled thiamine estimates to be 9–18 days (27). Excretion of thiamine is mainly by urine. Balance between renal loss and intestinal intake maintains the overall homeostasis, whereas other regulatory mechanisms ensure the stability in thiamine levels in the brain (2).

## Assessment of nutrient status

Plasma thiamine is the biomarker most responsive to thiamine intake (10). Other indicators of thiamine status include the ETKAC and by thiamine metabolites in accessible tissues. The ETKAC assay demonstrates actual functionality of the vitamin and is accordingly considered to be more informative than measuring the thiamine metabolites. The activity coefficient represents the degree of enzyme activity stimulation in vitro, and the activity of this enzyme depends on ThDP availability and on glucose phosphate availability. An activity coefficient below 1.15 is regarded as an indicator of sufficient status, and an activity coefficient of 1.15–1.25 indicates marginal status (30). The concentration of free thiamine and its phosphate esters in blood or erythrocytes has been shown to be a good indicator of thiamine status (31), especially among subjects at risk for thiamine deficiency (31, 32). The usefulness of the activity coefficient as an indicator of thiamine status in population surveys has been questioned, mainly due to its low correlation with erythrocyte thiamine (33).

## Dietary intake in Nordic and Baltic countries

Major food sources of thiamine in the Nordic diet are cereals and cereal products, meat and meat products, and milk and dairy products. Estimates of national dietary supply derive from dietary surveys in each country (see review on intake of vitamin and minerals in the Nordic countries). In the Nordic countries, the dietary supply is 1.4–1.5 mg thiamine/10 MJ except for Finland, where it is 2.0 mg/10 MJ based on an average that also takes into account the number of people in each age group. In the Baltic countries, the dietary supply is 1.2–1.4 mg thiamine/10 MJ.

## Health outcomes relevant for Nordic and Baltic countries

Chronic thiamine deficiency causes beriberi. In adults, symptoms include disturbances in the peripheral nervous system and heart function. Early deficiency symptoms can include anorexia, weight loss, mental changes and muscle weakness. Among children, symptoms appear more quickly, are generally more severe and can lead to heart failure. In alcoholics, conditions such as the Wernicke’s-Korsakoff’s syndrome can occur, and these are strongly related to insufficient thiamine intake and malabsorption (34, 35).

Thiamin also plays a part in refeeding syndrome, a metabolic disturbance following a period of starvation or metabolically stress from illness. Typically 3 to 7 days after refeeding starts, the plasma concentrations of phosphates, magnesium and potassium may fall. This disturbance may lead to neurological, cardiac or pulmonary malfunction, which, in severe cases, can be fatal. Thiamin treatment is usually part of the treatment of refeeding syndrome.

Several epidemiological studies have investigated the relationship between the intake of thiamine and other B-vitamins (folate, riboflavin and vitamins B_6_ and B_12_) and various cancers, particularly colorectal and breast cancer. No clear evidence for a relation between thiamine intake and different cancer forms has been found (36–38).

Thiamin has also been related to neurodegenerative disorders in the elderly such as Alzheimer’s disease (39), but evidence for a role in preventing neurological disorders is limited (40, 41). One systematic review on intake related to cognitive function concluded that evidence regarding higher thiamine intake and better cognitive function is weak (42).

The EU Scientific Committee for Food (28) concluded that it was not possible to set a safe upper intake level for thiamine due to a lack of data. Habitual thiamine intakes up to 6–7 mg/day have not been associated with negative effects. Oral intakes up to 500 mg/day for periods up to 1 month have not been associated with toxic effects. However, no health benefit has been identified with supplemental intake beyond the recommended amounts (43).

## Requirement and recommended intakes

The requirement of thiamine has been related to energy and carbohydrate intake (1, 44), and a clear relationship was shown by Sauberlich and co-workers (4). The current U.S. dietary reference intakes are, however, based on absolute intakes (45). Generally, thiamine intakes are related to energy and protein intakes in the normal intake ranges of populations such as those of the Nordic countries.

Clinical signs of deficiency have been observed at intakes below 0.5 mg/day, which corresponds to 0.05 mg/MJ (0.2 mg/1,000 kcal) (44, 45). In other studies, thiamine excretion in the urine and ETKAC were normalised at intakes of 0.07–0.08 mg/MJ (0.30–0.33 mg/1,000 kcal) (1).

The average requirements set in NNR 2012 (46) for adults and children were set at 0.10 mg/MJ, and the recommended intake was set at 0.12 mg/MJ. However, when planning diets with energy levels below 8 MJ/day, the thiamine content should be at least 0.8 mg/day. The recommended intake for infants 0–12 months old was set at 0.10 mg/MJ. The lower limit of intake was estimated at 0.05 mg/MJ.

Studies on pregnant and lactating women indicate a higher requirement as assessed by biochemical parameters (45). An additional intake of 0.4 mg/day during pregnancy and 0.5 mg/day during lactation is recommended.

A few studies indicate that thiamine utilisation is impaired among elderly subjects (47). Therefore, when planning diets for elderly with energy levels below 8 MJ/day, the thiamine content should be at least 1.0 mg/day.

## Conflict of interest and funding

None.
